# Flavored functional drinking yogurt (Doogh) formulated with *Lactobacillus plantarum* LS5, cress seed gum, and coriander leaves extract

**DOI:** 10.1002/fsn3.1367

**Published:** 2019-12-27

**Authors:** Zahra Shariati, Mohammad Jouki, Flora Rafiei

**Affiliations:** ^1^ Department of Food Science and Technology College of Agriculture Ashtian Branch Islamic Azad University Ashtian Iran; ^2^ Department of Food Science and Technology Faculty of Biological Sciences North Tehran Branch Islamic Azad University Tehran Iran

**Keywords:** antioxidant activity, coriander extract, functional properties, probiotic

## Abstract

The effects of coriander extract (CE) and cress seed gum (CSG) on viability of *Lactobacillus plantarum* LS5 and physicochemical properties of drinking yogurt (Doogh) were evaluated. The CE contained 18 mg GAE/g and was shown by the DPPH radical assay to have remarkable antioxidant activity. The CE was added at concentrations of 0%, 0.05%, 0.1%, and 0.25%, and the levels of added CSG were 0%, 0.1%, 0.25%, and 0.5%. Doogh samples were analyzed after 1, 2, and 3 weeks of storage at 4°C. By increasing the amounts of CSG, the viscosity of the Doogh samples was increased and phase separation was reduced significantly (*p* < .05). The results also showed that by increasing the levels of CSG to 0.5%, *L. plantarum* count increased significantly (*p* < .05). Doogh sample containing 0.05% CE and 0.5% CSG gained the highest probiotic count, overall acceptability score, and lowest lipid oxidation and phase separation in comparison with the other samples.

## Graphical abstract

To increase health benefits, dairy products can be formulated with probiotics and antioxidants for production of functional foods. The effects of coriander extract (CE) and cress seed gum (CSG) on viability of *Lactobacillus plantarum* LS5 and physicochemical properties of drinking yogurt (Doogh) were evaluated.

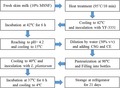

**Highlights**

- The coriander extract showed remarkable antioxidant activity and had 18 mg GAE/g.
- Probiotic Dooghs containing 0.5% of CSG were stable during storage period.
- Doogh formulated by 0.05% coriander extract showed higher survival probiotic rate.
- Combination of gum and extract at the suitable level prevented phase separation.
- Results indicated the potential of coriander extract for supporting probiotic growth.

## 1 INTRODUCTION

Drinking yogurt (Doogh) is a fermented beverage which is prepared by mixing yogurt with water and some salt and in some regions with herbal flavorings. Iran is one of the largest producers and consumers of Doogh in the world, with an annual production of 120.000 tons of Doogh produced from yogurt. It can be used instead of soda in the Iranian food basket, and it supplies a quarter of the daily requirement for calcium and contains vitamins B_2_, B_6_, and B_12_ (Foroughinaia, Abbasi, & Hamidi Esfahani, 2007). Doogh is a complex and heterogeneous system containing many different compounds such as proteins, lipids, and electrolytes. Therefore, to have a stable food system, the interaction between the components must be well balanced.

Edible gums are used as thickeners, stabilizers, gelling agents, emulsifiers or suspension stabilizers, and in some cases as prebiotic (Lucey, 2002). Among edible gums, cress seed gum (CSG) has shown a great potential to utilize as a stabilizing ingredient in the drinkable food systems. The functional properties of this gum have been extensively studied in many researches (Jouki, Khazaei, Ghasemlou, & HadiNezhad, 2013; Karazhiyan et al., 2009; Naji, Razavi, & Karazhiyan, 2012). Recently, Jouki et al. (2013) have shown that the CSG can be used as a gelling agent for the preparation of edible films. Cress seed gum is obtained from cress (*Lepidium sativum*) seeds when they soak in water and a transparent gel forms around the whole seed. Karazhiyan, Razavi, and Phillips (2011) reported that CSG, as a carbohydrate, contains sugars like l‐arabinose, d‐xylose, d‐galactose, l‐rhamnose, d‐glucose, d‐galacturonic acid, and 4‐O‐methyl‐d‐glucuronic acid.

Several studies use a number of polysaccharides like inulin, fructooligosaccharide, tara gum, guar gum, and gum arabic as prebiotics to enhance viability of probiotics (Akalin, Fenderya, & Akbulut, 2004; Gibson, Probert, Loo, Rastall, & Roberfroid, 2^2004^; Ranadheera, Baines, & Adams, 2010; Yilmaz‐Ersan, Ozcan, Akpinar‐Bayizit, & Omak, 2017). The prebiotic as a nondigestible food ingredient can usefully stimulate the growth or activity of living microorganisms that provide health advantages in the host colon (Gibson et al. (2004). Therefore, in this way, they can improve host health. Probiotics can be defined as living microorganisms that provide health advantages. As it has been reported, by beneficial effects of probiotics include improvement of the immune system, gastrointestinal complications, and lactose intolerance symptoms, as well as reduction of cholesterol and antitumor effects (El‐Abd et al., 2018). *Lactobacillus* and *Bifidobacterium* are the main genera of probiotic bacteria. To achieve better effects on host health, combining probiotics and natural prebiotics with Doogh may be helpful. As previously stated, plant extracts contain fibers, vitamins, and minerals that make it a suitable material to promote bacterial growth (El‐Abd et al., 2018).

Bioactive constituents, with antioxidant activity, are usually found in high concentrations in medical plants. Coriander (*Coriandrum sativum* L.) is a good source of natural antioxidants. It is a culinary and medicinal plant from the Umbelliferae family that is approved for food use by the FDA and can be used as an additive in food products and beverage (Mendel & Mandal, 2015). It is commonly utilized for its fresh leaves and the dry powder of its fruits which have organoleptic and flavoring properties (Yildiz, 2016). As has been reported by Burdock and Carabin (2009), this herb had been called the “spice of happiness” by the Egyptians because they considered it an aphrodisiac. As it has been shown by Shahwar et al. (2012), the methanol extract of coriander leaves has remarkable total phenolic contents (30.25 ± 3.42 mg/g) and the fresh leaves contain high values of carbohydrate, calcium, phosphorus, iron, vitamin B_2_, niacin, and vitamin C and vitamin A.

The heightened demands by consumers for better quality have given rise to the development of functional foods that support their health. The objective of the present work was to study the effect of CSG and coriander extract addition on physicochemical properties, *Lactobacillus plantarum* viability and stability of flavored Doogh during storage at refrigerator for 21 days.

## 2 METHODS AND MATERIALS

### 2.1 Materials

Fresh skim milk was provided from the Pegah Dairy Co. The milk contained 4.7% lactose, 1.4% fat, and 3.1% protein. A commercial strain of *L. plantarum* LS5 and lyophilized starter culture of *Streptococcus thermophiles* and *Lactobacillus delbrueckii* subsp. *bulgaricus* (YF‐3331) was obtained from Christian Hansen. The cress seeds and coriander leaves were obtained from the local market in Tehran, Iran, and all chemicals were obtained from Merck.

### 2.2 Preparation of cultures

*Lactobacillus plantarum* LS5 was activated in MRS broth medium and incubated at 37°C/24 hr under anaerobic conditions using an anaerobic atmosphere generation system (GasPak system, anaerobic system, Oxoid).

### 2.3 Extraction of gum from of cress seed

A sequential process was used to extract gum from cress seeds using the method described by Jouki et al. (2013). Briefly, aqueous CSG was extracted from whole seeds using distilled water (time 15 min, temperature 45°C, and water to seed ratio 30:1). The swelled seeds were stirred with a rod paddle blender at 1,100 to scrape the mucilage layer off the seed surface. The solutions were then filtered with cheese cloth, and the insoluble residue was filtered. The collected gum was dried in an oven (45°C overnight).

### 2.4 Preparation of coriander extract

Fresh coriander leaves were spread onto drying trays in freeze‐dryer (Biotron) at −55°C for 24 hr. Coriander extract was prepared by mixing 100 g of dried leaves with 300 ml of methanol then stirred at 25°C for 48 hr, and the extracts were filtered by Whatman No. 1 filter paper. To evaporate and remove the solvent from the extract, a rotary under vacuum was employed. The distillation was stopped when the volume of extract remaining was ~1 ml. Solvent was further removed under a purified N_2_ stream. The samples were kept under N_2_ in sealed vials in a freezer until use (Shahwar et al., 2012).

### 2.5 Determination of total phenolic content

The total phenolic contents of coriander extract determined by Folin–Ciocalteu method were reported to be gallic acid equivalents (GAE). Extract solution (125 μl) was taken in a volumetric flask, and 500 ml distilled water and 125 μl Folin–Ciocalteu reagent were added and shaken vigorously. Absorbance of the samples in triplicate at 760 nm was measured by using a UV‐vis spectrophotometer (Sun et al., 2006). Measurements were carried out in triplicate. Gallic acid was used at various concentrations (0–1,000 mg, 0.1 ml/L), and standard curve was obtained.

### 2.6 DPPH radical scavenging activity of coriander extract

The DPPH method is used for the determination of free radical scavenging activity, usually expressed as IC_50_ (Shahwar et al., 2012). Briefly, 1 ml of DPPH ethanol solution (freshly prepared at a concentration of 0.1 mM) was added to 3 ml of extract solution at various levels (10–100 μg/ml). The resulting mixture was stored in the dark place for 30 min at 20°C, and afterward, absorbance of samples was measured at 517 nm against ethanol. The scavenging activity of DPPH radicals was calculated according to the following equation:

(1) $$\text{Scavenging}\;\text{activity}\;\left(\%\right)=\frac{{\text{Abs}}_{\text{DPPH}}-{\text{Abs}}_{\text{sample}}}{{\text{Abs}}_{\text{DPPH}}}\times 100$$

The coriander extract concentration providing IC_50_ was measured using plotting concentrations against percentage inhibition. The amount of antioxidant needed to reduce the initial concentration of DPPH by 50% is defined as IC_50_, and lower amounts of IC_50_ indicate higher antioxidant activity.

### 2.7 Preparation of probiotic Doogh

**Figure 1:**
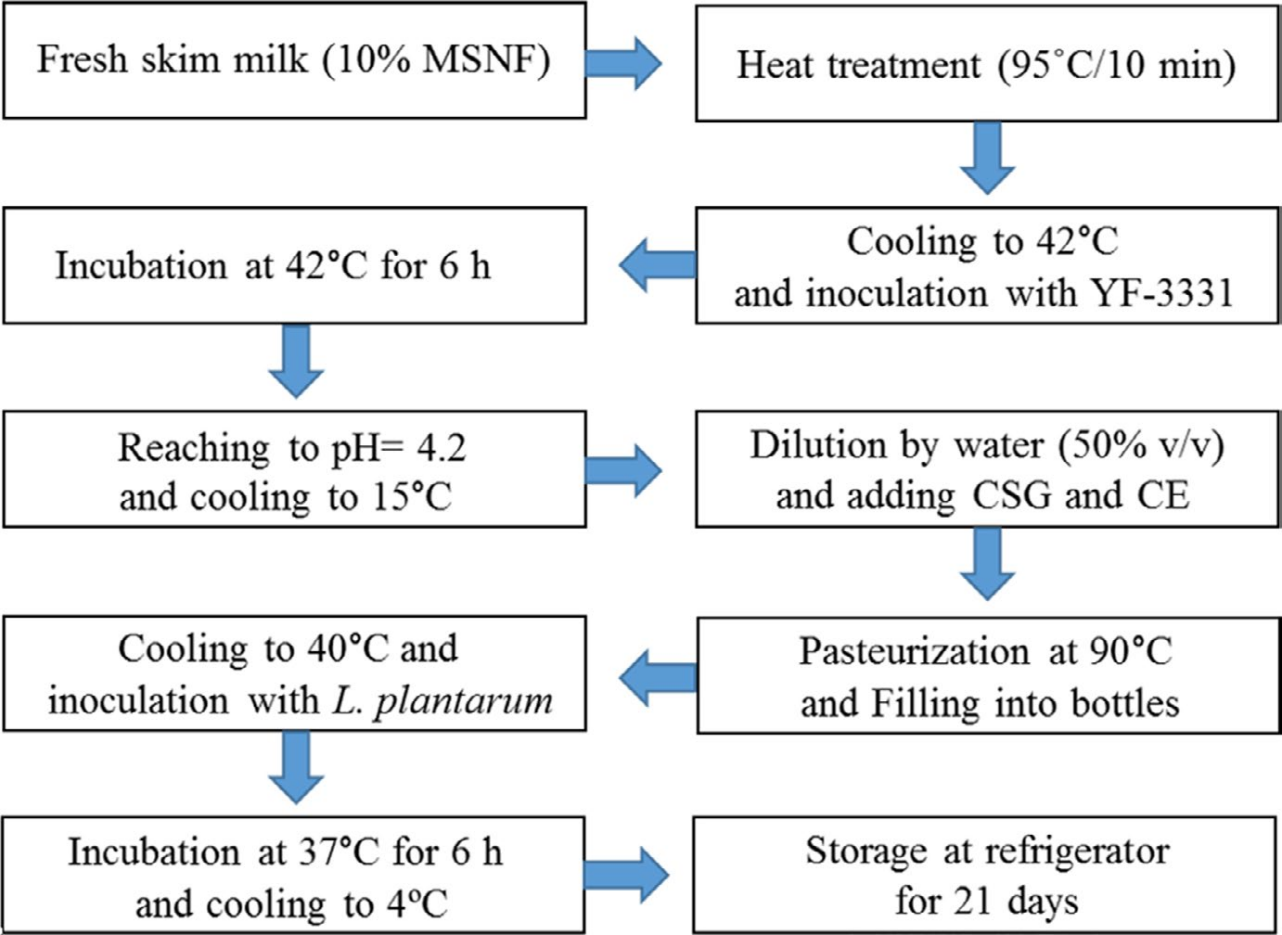
Functional Doogh production process flow chart

Fresh milk with 2.9% fat, 3.1% protein, 4.7% lactose, and pH 6.7 was pasteurized at 90°C for 15 min. After cooling down of the milk up to the fermentation temperature (42 ± 1°C), it was inoculated with YF‐3331 according to the manufacturer's instructions, incubated until its pH reached 4.2, and then cooled to 4°C to stop the fermentation. The yogurt was diluted by drinking water (50% v/v and NaCl 0.7% w/v) to produce Doogh. Then, CSG (0, 0.1, 0.25, and 0.5% w/v) and CE (0, 0.05, 0.1, and 0.25% w/v) were added to the mixture and stirred (Figure 1). Then, they were filled into 250‐ml PET bottles, and to achieve full hydration, the mixture was kept at room temperature overnight. All samples were pasteurized (90°C for 30 min) and cooled down to ambient temperature (Hashemi, Shahidi, Mortazavi, Milani, & Eshaghi, 2015). Afterward, active probiotic culture (10^9^ cfu/ml) was added to the Doogh samples and they were incubated at 37 ± 1°C for 6 hr. Then, Doogh samples were stored for 21 days at 4°C.

### 2.8 Microbial analysis

Viable counts of *L. plantarum* in Doogh samples were assessed by plate count method using MRS agar (Merck). The plates were incubated at 37°C for 72 hr in jars under anaerobic conditions (Hashemi et al., 2015). Microbiological colonies from the plates containing 30–300 colony‐forming units (cfu) were counted and transformed to log10 cfu/ml.

### 2.9 pH and titratable acidity

The pH changes of the Doogh samples were regularly measured at 25 ± 1°C using a HANNA pH meter. The titratable acidity (TA) was determined according to the method of Ghasempour, Alizadeh, and Bari (2012). Ten ml of Doogh samples and 10 ml of deionized water were mixed, and 0.5 ml of phenolphthalein was added into the mixture. This mixture was titrated with 0.1 N NaOH. The acidity of the Doogh samples was calculated as percent (%) lactic acid.

### 2.10 Phase separation

Phase separation of Doogh samples was measured using gravity separation. In order to determine stability of the Doogh samples, they were transferred to 10 ml sterilized and graduated test tubes with cap kept at 4°C. During 21 days of storage, the height of supernatant was measured and its value (divided by the total height of sample in bottle and multiplied by 100) has expressed as the serum separation (Foroughinaia et al., 2007).

### 2.11 Viscosity measurement

The dynamic viscosity of each Doogh samples was measured using a rotational viscometer (DV‐II, Brookfield Engineering Laboratories, Inc.). All measurements were done at room temperature (20°C) using a number 2 (LV2) spindle set at 30 rpm. After primary experiments, the appreciate torque value found between 10% and 100% of the measuring range to obtain reliable results.

### 2.12 Estimation of peroxide value in Doogh

The lipids were extracted from the Doogh samples according to the Bligh and Dyer's (1959) using chloroform/methanol (1:1 v/v). The peroxide value of the extracts was determined by the iodometric titration method described in the IUPAC standard method 2.501 (Paquot, 2013), and results were expressed in meq oxygen/kg lipid.

### 2.13 Sensory evaluation

Sensory evaluation was carried out by 9 trained panelists (including 5 women and 4 men, food science specialists, age 25–35) were asked to determine the sample scores with a 5‐point hedonic scale test. The scores (1 = dislike very much, 2 = dislike a little, 3 = neither like nor dislike, 4 = like a little, and 5 = like very much) for taste, color, odor, and overall acceptability were given by the expert panelists (Meilgaard, Civille, & Carr, 1999). Doogh samples were taken for the analysis after 1, 7, 14, and 21 days of storage.

### 2.14 Statistical analysis

The experimental data were statistically analyzed using SPSS statistical software. Duncan's multiple range tests were used to compare the differences among means. Also, the correlation coefficient analysis was done between the different parameters.

## 3 RESULTS AND DISCUSSION

### 3.1 Total phenolic content and antioxidant activity

The phenolic content of the coriander extract was 18.26 mg GAE/g, which agrees with the values found by Sriti, Aids Wannes, Talou, Vilarem, and Marzouk (2011) and Yildiz (2016) (15.16 and 14.973 mg GAE/g, respectively) but is lower than the 30.25 mg GAE/g found by Shahwar et al. (2012). The antioxidant activity of coriander extract was evaluated against the DPPH radical to measure the ability of the coriander extract to donate hydrogen to stabilize the radical, as assessed spectrophotometrically. In DPPH assay, IC_50_ value was 67.95 ± 0.08 μg/ml for ethanol extract of coriander extract. This result is in agreement with that of Yildiz (2016) although a potential less than 32 μg/ml was reported by Sriti et al. (2011). Yildiz (2016) has showed a higher free radical scavenging activity (IC_50_ of 74.87 ± 0.03 μg/ml) in comparison with our result (IC_50_ of 67.95 ± 0.08 μg/ml). Kahkonen et al. (1999) showed that antioxidant activity is directly related to total phenolic contents, so the sample having the higher total phenolic content will show the higher antioxidant activity. In addition, a significant correlation between antioxidant activity and total phenolic in some selected herbs was found by Wojdyło, Oszmian, and Czemerys (2007). In the other words, they reported that phenolic compounds are the dominant antioxidant components.

### 3.2 pH and acidity measurements

**Figure 2:**
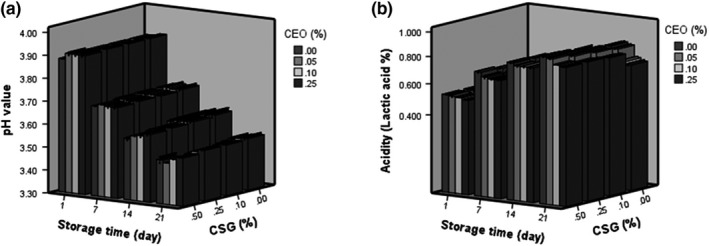
Effect of coriander extract (CE) and cress seed gum (CSG) concentrations on pH (a) and acidity (b) of probiotic Doogh during 21 days of storage

Statistical analysis showed that the effects of CSG gum and CE on the pH (Figure 2a) and acidity (Figure 2b) of probiotic Doogh were significant (*p* < .05). Doogh sample containing 0.5% gum and 0.05% CE had the highest acidity on the 21th day, and the lowest acidity was related to the sample containing 0% CSG and 0.25% CE. The pH of samples significantly decreased during the storage time, while the acidity increased (*p* < .05). Hashemi et al. (2015) reported that the increase in acidity in Doogh when inulin was applied. Kneifel, Jaros, and Erhard (1993) reported a similar trend for yogurt samples was investigated. According to their results, titratable acidity of the samples increased during storage at 6°C. As it has been reported by several researchers, this increase could be due to the production of lactic acid and other organic acids by lactic cultures (Bakirci & Kavaz, 2008; Joung et al., 2016).

**Figure 3:**
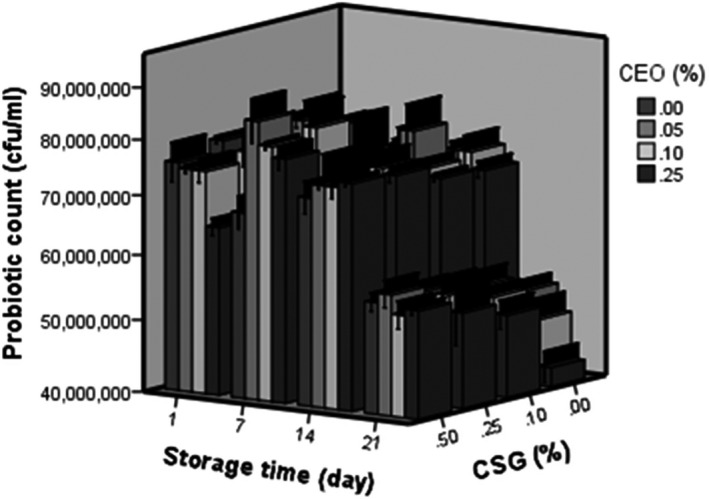
Effect of coriander extract (CE) and cress seed gum (CSG) concentrations on probiotic viability of Doogh during 21 days of storage

The results of this part were consistent with the obtained results of probiotic viability (Figure 3). As shown in Figure 3, the addition of CE at 0.05% increased probiotic viability, and increase in probiotic counts in the presence of CE might be due to the presence of prebiotic compounds including phenolic compounds. As El‐Abd et al. (2018) stated plant extracts contain fibers, vitamins, and minerals that make it a suitable material to promote bacterial growth. Therefore, in this study, reduction in pH and increase in acidity were higher in the samples containing 0.05% CE and high level of CSG because of the bacterial growth and production of lactic acid. Moreover, the acidity slightly decreased at 0.1% and 0.25% of CE compared to 0.25%. This could be related to antimicrobial activity of CE at higher level due to the presence of some phenolic and antibacterial components in CE.

### 3.3 Survival of *L. plantarum*

Figure 3 shows that the effects of storage time, the concentration of CSG and CE effects on survival of *L. plantarum* are significant (*p* < .05). As results show, the cell numbers of *L. plantarum* were significantly reduced over 21 days of storage. These results are consistent with the results presented by Hashemi et al. (2015), who showed that the *Lactobacillus paracasei* count decreased significantly during storage time, although the probiotic population in yogurt was above 7 log CFU/g, after 2 weeks of storage. The number of *L. plantarum* was significantly (*p* ˂ .05) increased by addition of 0.25% of CSG, but increasing the level of CSG to more than this amount had no significant effect (*p* ≥ .05). Ziaolhagh and Jalali, (2017), reported that xanthan gum could stimulate the growth of *Bifidibacterium lactis* in Doogh samples. It has been reported that the population of *Lactobacillus rhamnosus* as a probiotic in the food product containing carrageenan–maltodextrin was higher compared to the control sample (Karlton‐Senaye, Tahergorabi, Giddings, & Ibrahim, 2015). These results thus indicate that this CSG could maintain the viability of probiotic bacteria. Doogh samples fortified with CSG showed good viability at the end of the storage period compared to the control sample.

As reported by Karlton‐Senaye and Ibrahim (2013), some gums have good source of nutrients for bacterial growth can act as prebiotic and stimulate the growth of lactic acid bacteria. Our results also revealed that addition of 0.05% of coriander extract increased the survival rate of *L. plantarum* in Doogh samples. These results are consistent with the findings of Haddadin (2010), who found increase in the viability of probiotics in the presence of olive leaf extracts due to prebiotic compounds including phenolic compounds. Haji Ghafarloo, Jouki, and Tabari (2019) stated that ginger extract at low concentration acts as prebiotic to promote the *Bifidobacterium bifidum* growth in Doogh sample. As Shahwar et al. (2012) reported, the coriander leaves contain remarkable values of carbohydrates and water soluble vitamins. Therefore, the coriander extract can improve the viability of the *L. plantarum* during cold storage because they are able to supply additional nutrients for increasing culture growth.

At the end of the storage time, probiotic viability was slightly better in samples enriched by CE compared to the control. So, these results are consistent with the findings of El‐Abd et al. (2018), who found increase in the viability of probiotics in the presence of ginger aqueous extract (GAE) due to prebiotic compounds including phenolic compounds. El‐Abd et al. (2018) reported that the addition of ginger aqueous extract (GAE) increased bacterial counts of probiotic strains in fermented camel milk.

### 3.4 Viscosity of Doogh

**Figure 4:**
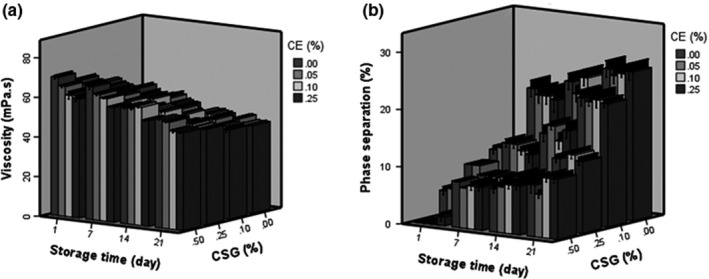
Effect of coriander extract (CE) and cress seed gum (CSG) concentrations on viscosity (a) and phase separation (b) of probiotic Doogh during 21 days of storage

Storage time affected viscosity of Doogh samples, negatively (*p* ˂ .05). It can be concluded that the addition of CE causes a decrease in viscosity and consistency of the Doogh sample (Figure 4a). As it is shown in Figure 4a, the viscosity of probiotic Doogh was increased significantly (*p* ˂ .05) as the concentration of CSG increased from 0% to 0.5%. As it has been reported by Koksoy and Kilic (2004) and Paraskevopoulou et al. (2003), the viscosity of dairy drink is changed in the presence of gums. Ziaolhagh and Jalali (2017) reported that xanthan gum increased the viscosity of bio‐Doogh, and Paraskevopoulou et al. (2003) showed that it can increase the viscosity of a daily drink made from kefir–milk mixture. The viscosity of the samples decreased significantly (*p* < .05) during storage time. This decrease might be due to microbial enzyme action on the casein micelle matrix during the storage period (Kosikowski, 1982).

### 3.5 Phase separation

Figure 4b also shows that phase separation was decreased significantly during storage, agreeing with the observations by Hashemi et al. (2015). Moreover, increasing the concentration of CE accelerated the phase separation in Doogh during storage period. Results showed that phase separation was lower in all treated samples compared to control during 21 days of storage. As Figure 4b shown, addition of CSG even at low concentration (0.25%) improved the prevention of phase separation in Doogh samples, and the stability of Doogh samples was increased by increasing in CSG content from 0.25% to 0.5% (Figure 4b). The CSG used in this study, as food stabilizer, prevented phase separation in Doogh samples. It could be due to its ability to enhance the viscosity and bind water. Phase separation is a common phenomenon taking place in acid dairy drinks, and several researchers have used stabilizers to prevent phase separation in Doogh (Azarikia & Abbasi, 2010; Koksoy & Kilic, 2003; Ziaolhagh & Jalali, 2017). Reduction of the phase separation as a result of gums presence was related to the weak gel network created with incorporation of gums. Ziaolhagh and Jalali (2017) studied the effects of wild thyme essence and xanthan gum on phase separation of bio‐Doogh.

### 3.6 Peroxide value (PV)

Primary lipid oxidation was evaluated by means of PV (Jouki, Mortazavi, Tabatabaei Yazdi, Koocheki, & Khazaei, 2014). As it has been stated, chemical changes caused by lipid oxidation are the main sources of undesirable flavors in food products. Peroxide formation through the spontaneous reaction of oxygen with unsaturated fatty acids followed by the generation of carbonyl compounds is one of these changes (O'Conner & O'Brien, 2006). The effect of treatments and storage time on changes of PV of Doogh samples is depicted in Table 1. The initial PV (meq peroxide/kg) in the analyzed Doogh samples ranged from 0.13 to 0.16. At the first day of storage time, no significant difference (*p* > .05) was observed among different samples. The PV significantly increased for all samples during storage time, indicating that lipid oxidation is continued under the storage conditions (*p* < .05). As we expected, a positive and high correlation was observed between PV and pH. The PV of the control and treated samples without extract (with a pH 3.69–3.71) increased significantly from an initial value of 0.13–0.16 meq/kg until day 7 and eventually reached to 0.17–0.20 meq/kg (Table 1). The samples containing coriander extract showed lower rate of peroxide formation, which have higher pH. This may be attributed to the higher rate of lipid auto‐oxidation at an acidic pH and thus to the more pro‐oxidant environment of pH 3.5 (day 21) in comparison with pH 3.7 (day 1). This result is in agreement with that of Sasanian et al. (2018) on drinking yoghurt. As has been reported by Mei, McClements, and Decker (1999), when the pH decreases to the protein isoelectric point, it may influence positively charged proteins, which can repel positively charged ions (Cu^2+^, Fe^3+^) that subsequently accelerate lipid oxidation. The PV of samples treated with CE was significantly lower than that of untreated by CE, indicating that the coriander extract was effective in reducing lipid oxidation in Doogh (Table 1). The results also showed that the highest level of coriander extract (0.25%) had the highest effect in slowing down the primary peroxidation process, compared with other samples (*p* < .05). As has been previously reported by Shahwar et al. (2012) and Yildiz (2016), coriander extract showed good antioxidant activity. Perumalla and Hettiarachchy (2011) showed that the antioxidant activity of the plant extracts could be related to mechanisms such as inhibition of radical chain initiation, decomposition of peroxides, and interaction with the free radicals as well as binding of metal ion catalysts connection. On day 21 of storage, PV of samples enriched with 0.25% coriander extract was 17% lower than control sample (Table 1). Meanwhile, PVs of all Doogh samples were lower than 1 meq O_2_/kg of oil until day 21 of storage. In addition, in present study the samples were stored in a dark refrigerator and were protected from light. These conditions most likely contributed to the inhibition of lipid oxidation even at the lowest concentration coriander extract (0.05%).

**Table 1:** Effect of treatments and storage time on peroxide value (PV) (meq O_2_/kg fat)

Samples	CSG (%)	CE (%)	Day 1	Day 7	Day 14	Day 21
T1	0	0	0.14 ± 0.01a,C	0.20 ± 0.02a,B	0.34 ± 0.02a,A	0.31 ± 0.02ab,A
T2	0	0.05	0.15 ± 0.02a,B	0.19 ± 0.03a,B	0.34 ± 0.04a,A	0.32 ± 0.02ab,A
T3	0	0.1	0.13 ± 0.03a,B	0.17 ± 0.02ab,B	0.29 ± 0.03ab,A	0.30 ± 0.01ab,A
T4	0	0.25	0.14 ± 0.01a,B	0.15 ± 0.02b,B	0.30 ± 0.04ab,A	0.28 ± 0.03b,A
T5	0.1	0	0.14 ± 0.02a,C	0.22 ± 0.02a,B	0.34 ± 0.03a,A	0.34 ± 0.02a,A
T6	0.1	0.05	0.14 ± 0.01a,D	0.19 ± 0.03a,C	0.29 ± 0.03ab,B	0.35 ± 0.02a,A
T7	0.1	0.1	0.14 ± 0.02a,B	0.16 ± 0.02b,B	0.27 ± 0.04b,A	0.30 ± 0.02ab,A
T8	0.1	0.25	0.13 ± 0.01a,C	0.17 ± 0.02b,B	0.26 ± 0.02b,A	0.28 ± 0.03b,A
T9	0.25	0	0.15 ± 0.02a,C	0.20 ± 0.02a,B	0.33 ± 0.02a,A	0.35 ± 0.03a,A
T10	0.25	0.05	0.13 ± 0.01a,C	0.18 ± 0.03aB	0.31 ± 0.03a,A	0.33 ± 0.02ab,A
T11	0.25	0.1	0.14 ± 0.02a,C	0.18 ± 0.02a,C	0.27 ± 0.03b,B	0.34 ± 0.01a,A
T12	0.25	0.25	0.13 ± 0.02a,C	0.15 ± 0.02b,C	0.25 ± 0.02b,B	0.30 ± 0.02ab,A
T13	0.5	0	0.16 ± 0.02a,C	0.22 ± 0.02a,B	0.35 ± 0.03a,A	0.33 ± 0.01a,A
T14	0.5	0.05	0.15 ± 0.02a,B	0.17 ± 0.03ab,B	0.32 ± 0.01a,A	0.30 ± 0.02b,A
T15	0.5	0.1	0.14 ± 0.01a,B	0.18 ± 0.03ab,B	0.30 ± 0.03ab,A	0.30 ± 0.03ab,A
T16	0.5	0.25	0.13 ± 0.02a,C	0.16 ± 0.02b,C	0.27 ± 0.02b,B	0.27 ± 0.02b,A

Note

Means within each column followed by different letters (a‐d) show significant different (*p* < .05) between treatments at the same time. Means within each row followed by different letters (A‐D) show significant different (*p* < .05) at a treatment during storage period.

### 3.7 Sensory characteristics

**Figure 5:**
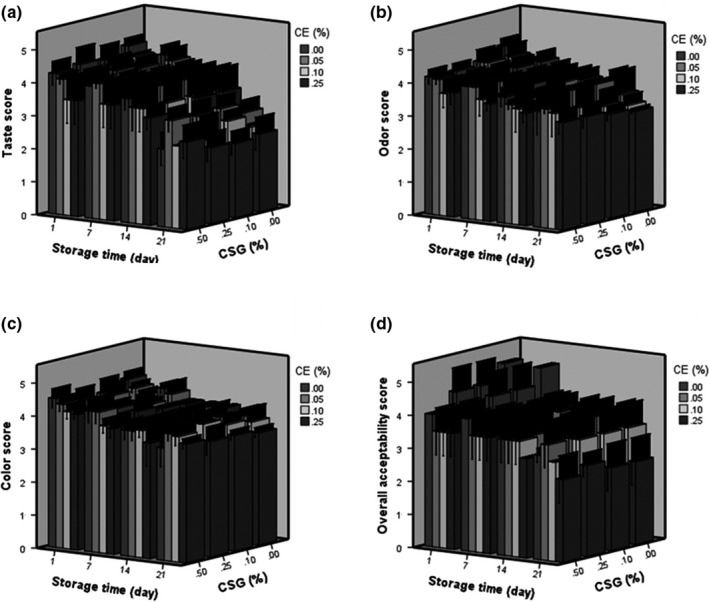
Effect of coriander extract (CE) and cress seed gum (CSG) concentrations on sensory scores of Doogh samples during 21 days of storage. Taste (a), odor (b), color (c), and overall acceptability (d)

As it is shown in Figure 5, the effect of concentrations of CSG and CE on the sensory attributes of the Doogh samples at the first day of storage was significant (*p* ˂ .05). The sensory evaluation of samples showed that the concentration of CSG and CE had significant (*p* < .05) effects on the taste, color, odor, and overall acceptability of probiotic Doogh samples (Figure 5a–d). The panelists preferred probiotic Doogh containing 0.05% of CE. Higher levels of CE caused a bitter taste. In addition, higher amount of CE can significantly decrease the stability by increasing the phase separation. However, the panelist could not identify the differences in the odor and color between control and the sample enriched with 0.05% CE + 0.5%CSG.

The control had the lowest taste and odor scores at the end of storage. As has been reported by Dave and Shah (1997), producing the fermented dairy products only by probiotic bacteria leads to the unfavorable tasted products. So, the gums used in this study could cover the unfavorable sensory attributes in Doogh samples. The higher scores were related to the Doogh sample containing 0.05% CE at the end of storage. Azarikia and Abbasi (2010) showed that the presence of local herbs extracts in Doogh stabilized with tragacanthin could enhance its taste score. As illustrated in the Figure 5a, the panelists evaluated that Doogh stabilized by 0.5% CSG and 0.05% CE can present the best taste among the other samples. This sample also had a high color and odor score (Figure 5). The panelists evaluated that the prepared samples with high CSG concentration (have highest viscosity) are ideal for the consumers. Penna, Sivieri, and Oliviera (2001) stated that the sensory acceptability of lactic beverages can be increased by increasing the viscosity.

## 4 CONCLUSION

The addition of CSG and CE to Doogh significantly affected physicochemical properties including pH, titratable acidity (LA %), viscosity, phase separation, lipid oxidation, and sensorial properties of Doogh. The results of this study showed that probiotic Doogh samples containing 0.5% CSG and 0.05% CE maintained the quality of Doogh samples by preventing color changes and lipid oxidation and phase separation as well as other quality parameters. Therefore, coriander extract as a natural antioxidant can be added to dairy products to effectively inhibit oxidation during storage.

## CONFLICT OF INTEREST

We declare no potential conflict of interest related to this manuscript.

## ETHICAL APPROVAL

We declare no ethical issue related with this article.

## References

[fsn31367-bib-0001] Akalin, A. S. , Fenderya, S. , & Akbulut, N. (2004). Viability and activity of bifidobacteria in yoghurt containing fructooligosaccharide during refrigerated storage. International Journal of Food Science and Technology, 39, 613–621. 10.1111/j.1365-2621.2004.00829.x

[fsn31367-bib-0002] Azarikia, F. , & Abbasi, S. (2010). On the stabilization mechanism of Doogh (Iranian yoghurt drink) by gum tragacanth. Food Hydrocolloids, 24, 358–363. 10.1016/j.foodhyd.2009.11.001

[fsn31367-bib-0003] Bakirci, I. , & Kavaz, A. (2008). An investigation of some properties of banana yogurts made with commercial ABT‐2 starter culture during storage. International Journal of Dairy Technology, 61, 270–276. 10.1111/j.1471-0307.2008.00409.x

[fsn31367-bib-0004] Bligh, E. G. , & Dyer, W. J. (1959). A rapid method of total lipid extraction and purification. Canadian Journal of Biochemistry and Physiology, 37, 911–917. 10.1139/y59-099 13671378

[fsn31367-bib-0005] Burdock, G. A. , & Carabin, I. G. (2009). Safety assessment of coriander (*Coriandrum sativum* L.) essential oil as a food ingredient. Food and Chemical Toxicology, 47, 22–34. 10.1016/j.fct.2008.11.006 19032971

[fsn31367-bib-0006] Dave, R. Z. , & Shah, N. P. (1997). Viability of yogurt and probiotic bacteria in yogurts made from commercial starter cultures. International Dairy Journal, 7, 31–41.

[fsn31367-bib-0007] El‐Abd, M. M. , Abdel‐Hamid, M. , El‐ Sayed, H. S. , El‐ Metwaly, H. A. , El‐Demerdash, M. E. , & Mohamed, Z. F. A. (2018). Viability of microencapsulated probiotics combined with plant extracts in fermented camel milk under simulated gastrointestinal conditions. Middle East Journal of Applied Sciences, 8, 837–850.

[fsn31367-bib-0008] Foroughinaia, S. , Abbasi, S. , & Hamidi Esfahani, Z. (2007). Effects of individual and combined addition of salep, tragacantin and guar gums on the stabilization of Iranian Doogh. Iranian Journal of Nutrition Sciences & Food Technology, 2, 15–27.

[fsn31367-bib-0009] Ghasempour, Z. , Alizadeh, M. , & Bari, M. R. (2012). Optimization of probiotic yogurt production containing Zedo gum. International Journal of Dairy Technology, 65, 118–125.

[fsn31367-bib-0010] Gibson, G. R. , Probert, H. M. , Loo, J. V. , Rastall, R. A. , & Roberfroid, M. B. (2004). Dietary modulation of the human colonic microbiota: Updating the concept of prebiotics. Nutrition Research Reviews, 17, 259–275. 10.1079/NRR200479 19079930

[fsn31367-bib-0011] Haddadin, M. S. Y. (2010). Effect of olive leaf extracts on the growth and metabolism of two probiotic bacteria of intestinal origin. Pakistan Journal of Nutrition, 9, 787–879. 10.3923/pjn.2010.787.793

[fsn31367-bib-0012] Haji Ghafarloo, M. , Jouki, M. , & Tabari, M. (2019). Production and characterization of synbiotic Doogh, a yogurt‐based Iranian drink by gum arabic, ginger extract and *B. bifidum* . Journal of Food Science and Technology, 10.1007/s13197-019-04151-4 PMC7026337 32123437

[fsn31367-bib-0013] Hashemi, S. M. B. , Shahidi, F. , Mortazavi, S. A. , Milani, E. , & Eshaghi, Z. (2015). Synbiotic potential of Doogh supplemented with free and encapsulated *Lactobacillus plantarum* LS5 and *Helianthus tuberosus* inulin. Journal of Food Science and Technology, 52, 4579–4585. 10.1007/s13197-014-1511-7 26139928 PMC4486574

[fsn31367-bib-0014] Jouki, M. , Khazaei, N. , Ghasemlou, M. , & HadiNezhad, M. (2013). Effect of glycerol concentration on edible film production from cress seed carbohydrate gum. Carbohydrate Polymers, 96, 39–46. 10.1016/j.carbpol.2013.03.077 23688452

[fsn31367-bib-0015] Jouki, M. , Mortazavi, S. A. , Tabatabaei Yazdi, F. , Koocheki, A. , & Khazaei, N. (2014). Use of quince seed mucilage edible films containing natural preservatives to enhance physico‐chemical quality of rainbow trout fillets during cold storage. Food Science and Human Wellness, 3, 65–72. 10.1016/j.fshw.2014.05.002

[fsn31367-bib-0016] Joung, J. Y. , Lee, J. Y. , Ha, Y. S. , Shin, Y. K. , Kim, Y. , Kim, S. H. , & Oh, N. S. (2016). Enhanced microbial, functional and sensory properties of herbal yogurt fermented with Korean traditional plant extracts. Korean Journal for Food Science of Animal Research, 36(1), 90–99. 10.5851/kosfa.2016.36.1.90 PMC4973947 27499669

[fsn31367-bib-0017] Kähkönen, M. P. , Hopia, A. I. , Vuorela, H. J. , Rauha, J.‐P. , Pihlaja, K. , Kujala, T. S. , & Heinonen, M. (1999). Antioxidant activity of plant extracts containing phenolic compounds. Journal of Agricultural and Food Chemistry, 47, 3954–3962. 10.1021/jf990146l 10552749

[fsn31367-bib-0018] Karazhiyan, H. , Razavi, S. M. A. , & Phillips, G. O. (2011). Extraction optimization of a hydrocolloid extract from cress seed (*Lepidium sativum*) using response surface methodology. Food Hydrocolloids, 25, 915–920. 10.1016/j.foodhyd.2010.08.022

[fsn31367-bib-0019] Karazhiyan, H. , Razavi, S. M. A. , Phillips, G. O. , Fang, Y. , Al‐Assaf, S. , Nishinari, K. , & Farhoosh, R. (2009). Rheological properties of *Lepidium sativum* seed extract as a function of concentration, temperature and time. Food Hydrocolloids, 23, 2062–2068. 10.1016/j.foodhyd.2009.03.019

[fsn31367-bib-0020] Karlton‐Senaye, B. D. , & Ibrahim, S. A. (2013). Impact of gums on the growth of probiotics. Agro Food Industries: Functional Food, Nutraceutical, 24, 10–14.

[fsn31367-bib-0021] Karlton‐Senaye, B. D. , Tahergorabi, R. , Giddings, V. L. , & Ibrahim, S. A. (2015). Effect of gums on viability and ‐galactosidase activity of *Lactobacillus* spp. in milk drink during refrigerated storage. International Journal of Food Science and Technology, 50, 32–40.

[fsn31367-bib-0022] Kneifel, W. , Jaros, D. , & Erhard, F. (1993). Microflora and acidification properties of yogurt and yogurt‐related products fermented with commercially available starter cultures. International Journal of Food Microbiology, 18, 179–189. 10.1016/0168-1605(93)90043-G 8494687

[fsn31367-bib-0023] Koksoy, A. , & Kilic, M. (2003). Effect of water and salt level on rheological properties of Ayran, a Turkish yoghurt drink. International Dairy Journal, 13, 835–839.

[fsn31367-bib-0024] Koksoy, A. , & Kilic, M. (2004). Use of hydrocolloids in textural stabilization of a yoghurt drink, ayran. Food Hydrocolloids, 18, 593–600. 10.1016/j.foodhyd.2003.10.002

[fsn31367-bib-0025] Kosikowski, F. V. (1982). Cheese and fermented milk foods (2nd ed.). Ithaca, IL: Edwards Brothers, Inc.

[fsn31367-bib-0026] Lucey, J. A. (2002). Formation and physical properties of milk protein gels. Journal Dairy Science, 85, 281–294. 10.3168/jds.S0022-0302(02)74078-2 11913691

[fsn31367-bib-0027] Mei, L. , McClements, D. J. , & Decker, E. A. (1999). Lipid oxidation in emulsions as affected by charge status of antioxidants and emulsion droplets. Journal of Agricultural and Food Chemistry, 47, 2267–2273. 10.1021/jf980955p 10794621

[fsn31367-bib-0028] Meilgaard, M. , Civille, G. V. , & Carr, B. T. (1999). Selection and training of panel members In: Meilgaard M. C., Carr B. T., & Civill G. V. (Eds.), Sensory evaluation techniques (pp. 133–158). Boca Raton, FL: CRC Press Inc.

[fsn31367-bib-0029] Mendel, S. , & Mandal, M. (2015). Coriander (*Coriandrum sativum* L.) essential oil: Chemistry and biological activity. Asian Pacific Journal of Tropical Biomedicine, 5, 421–442.

[fsn31367-bib-0030] Naji, S. , Razavi, S. M. A. , & Karazhiyan, H. (2012). Effect of thermal treatment on functional properties of cress seed (*Lepidium sativum*) and xanthan gums: A comparative study. Food Hydrocolloids, 28, 75–81.

[fsn31367-bib-0031] O'Conner, T. P. , & O'Brien, N. M. (2006). Lipid oxidation In Fox P. F., & McSweeney P. L. H. (Eds.), Advanced dairy chemistry: Lipids (Volume 2, pp. 558–560). New York, NY: Springer.

[fsn31367-bib-0032] Paquot, C. (2013). Standard methods for the analysis of oils, fats and derivatives (6th ed.). Great Britain: Elsevier Science.

[fsn31367-bib-0033] Paraskevopoulou, A. , Athanasiadis, I. , Blekas, G. , Koutinas, A. A. , Kanellaki, M. , & Kiosseoglou, V. (2003). Influence of polysaccharide addition on stability of a cheese whey kefir‐milk mixture. Food Hydrocolloids, 17, 615–620. 10.1016/S0268-005X(02)00122-4

[fsn31367-bib-0034] Penna, A. L. B. , Sivieri, K. , & Oliviera, M. N. (2001). Relation between quality and rheological properties of lactic beverages. Journal of Food Engineering, 49, 7–13. 10.1016/S0260-8774(00)00179-5

[fsn31367-bib-0035] Perumalla, A. V. S. , & Hettiarachchy, N. S. (2011). Green tea and grape seed extracts—Potential applications in food safety and quality. Food Research International, 44, 827–839. 10.1016/j.foodres.2011.01.022

[fsn31367-bib-0036] Ranadheera, R. D. C. S. , Baines, S. K. , & Adams, M. C. (2010). Importance of food in probiotic efficacy. Food Research International, 43, 1–7. 10.1016/j.foodres.2009.09.009

[fsn31367-bib-0037] Sasanian, N. , Mortazavian, A. M. , Hosseini, H. , Mohammadi, R. , Nayebzadeh, K. , & Sasanian, N. (2018). Development of traditional flavour in commercial doogh by addition of lipase. International Journal of Dairy Technology, 70, 1–10. 10.1111/1471-0307.12528

[fsn31367-bib-0038] Shahwar, M. K. , El‐Ghorab, A. H. , Anjum, F. M. , Butt, M. S. , Hussain, S. , & Nadeem, M. (2012). Characterization of coriander (*Coriandrum sativum* L.) seeds and leaves: Volatile and nonvolatile extracts. International Journal of Food Properties, 15, 736–747.

[fsn31367-bib-0039] Sriti, J. , Aids Wannes, W. , Talou, T. , Vilarem, G. , & Marzouk, B. (2011). Chemical composition and antioxidant activities of Tunisian and Canadian coriander (*Coriandrum sativum* L.) fruit. Journal of Essential Oil Research, 8, 7–15.

[fsn31367-bib-0040] Sun, T. , Xu, Z. , Wu, C. T. , Janes, M. , Prinyawiwatkul, W. , & No, H. K. (2006). Antioxidant activities of different colored sweet bell pepper (*Capsicum annum* L.). Journal of Food Science, 72, 98–102. 10.1111/j.1750-3841.2006.00245.x 17995862

[fsn31367-bib-0041] Wojdyło, A. , Oszmian, J. , & Czemerys, R. (2007). Antioxidant activity and phenolic compounds in 32 selected herbs. Food Chemistry, 105, 940–949. 10.1016/j.foodchem.2007.04.038

[fsn31367-bib-0042] Yildiz, H. (2016). Chemical composition, antimicrobial, and antioxidant activities of essential oil and ethanol extract of *Coriandrum sativum* L. leaves from Turkey. International Journal of Food Properties, 19, 1593–1603.

[fsn31367-bib-0043] Yilmaz‐Ersan, L. , Ozcan, T. , Akpinar‐Bayizit, A. , & Omak, G. (2017). Impact of some gums on the growth and activity of *Bifidobacterium animalis* subsp. *lactis* . International Journal of Food Engineering, 3, 73–77. 10.18178/ijfe.3.1.73-77

[fsn31367-bib-0044] Ziaolhagh, S. H. , & Jalali, H. (2017). Physicochemical properties and survivability of probiotics in bio‐Doogh containing wild thyme essence and xanthan gum. International Food Research Journal, 24, 1805–1810.

